# Perflutren lipid microspheres for echocardiogram contrast: a clinical case of anaphylaxis not caused by PEG

**DOI:** 10.1186/s13223-025-00964-5

**Published:** 2025-05-07

**Authors:** Chloe Wang, Jackie Campbell, Harriet Lea-Banks, Erika Lee

**Affiliations:** 1 Faculty of Science and Schulich School of Medicine & Dentistry, Western University, London, ON Canada; 2 Drug Allergy Clinic, Sunnybrook Health Sciences Centre, Toronto, ON Canada; 3 Physical Sciences Platform, Sunnybrook Research Institute, Toronto, ON Canada; 4 Department of Medical Imaging, Temerty Faculty of Medicine, University of Toronto, Toronto, ON Canada; 5 Division of Clinical Immunology & Allergy, Department of Medicine, University of Toronto, Toronto, ON Canada

**Keywords:** Perflutren lipid microspheres, Ultrasound contrast agent, Echocardiogram contrast agent, Anaphylaxis, PEG, Polyethylene glycol, IgE-mediated reaction, Complement activation-related pseudoallergy

## Abstract

**Background:**

Perflutren lipid microsphere suspension, sold under the brand name Definity®, is a microbubble ultrasound contrast agent. The microspheres contain octafluoropropane (C_3_F_8_) gas encapsulated by an outer lipid shell of phospholipids and a polyethylene glycol (PEG)ylated phospholipid. Anaphylaxis to perflutren lipid microsphere is very rare, with only one case report clearly attributing the reaction to the PEG excipient. We report a novel case of anaphylaxis likely caused by a non-PEGylated component of Definity®.

**Case presentation:**

Our patient is a healthy 54-year-old female, who underwent an exercise stress transthoracic echocardiogram using Definity® as an enhancing agent. She experienced anaphylaxis within 15 min of injection. Symptoms resolved after she was treated with diphenhydramine and epinephrine, followed by a systemic corticosteroid and ondansetron in the Emergency Department. The patient underwent allergy testing at our clinic for Definity® and various PEG-containing substances. While all PEG products tested negative, she had positive intradermal tests to Definity®. She also had negative skin prick testing to PEG 8000 and passed an oral challenge to PEG 3350, thus ruling out PEG as the causative agent of anaphylaxis.

**Conclusions:**

Our case report highlights a previously undocumented instance of anaphylaxis to Definity® not caused by PEG. We suspect the reaction to be an IgE-mediated response to a non-PEGylated component of Definity®. An alternative explanation for the reaction could be a complement activation-related pseudoallergy. This report provides critical information to physicians on the potential risks of using Definity® and contributes to growing research surrounding the profile of Definity®.

## Background

Perflutren lipid microsphere suspension, sold under the brand name Definity®, is a microbubble ultrasound contrast agent (UCA) widely used across North America. Injected intravenously, its physical acoustic properties allow delineation of the inner border of the left ventricle of the heart, which clarifies images during echocardiograms. Upon activation, the microspheres are made of octafluoropropane (C_3_F_8_) gas encapsulated by an outer lipid shell, consisting of phospholipids and a PEGylated phospholipid (MPEG5000 DPPE) with an approximate molecular weight (MW) of 5750 [1, 2]. Anaphylactoid reactions to Definity® are very rare [3] and only one case report of anaphylaxis was clearly attributed to polyethylene glycol (PEG) [4].

## Case presentation

We report a novel case of anaphylaxis to the non-PEGylated component of Definity®. Our patient is a healthy 54-year-old female, who underwent an exercise stress transthoracic echocardiogram at her cardiologist’s office, using Definity® as an enhancing agent. Within 15 min after injection and while walking on a treadmill, she developed urticaria on her arms that spread to the torso, followed by syncope. Upon arousal, she experienced emesis, urinary incontinence, tongue swelling, and dyspnea. According to a report from the patient’s husband, she was hypotensive before paramedic arrival. She was treated with diphenhydramine intravenously, and paramedics later gave her epinephrine. She also received a systemic corticosteroid and ondansetron while in the emergency department, where her symptoms resolved after several hours.

**Fig. 1:**
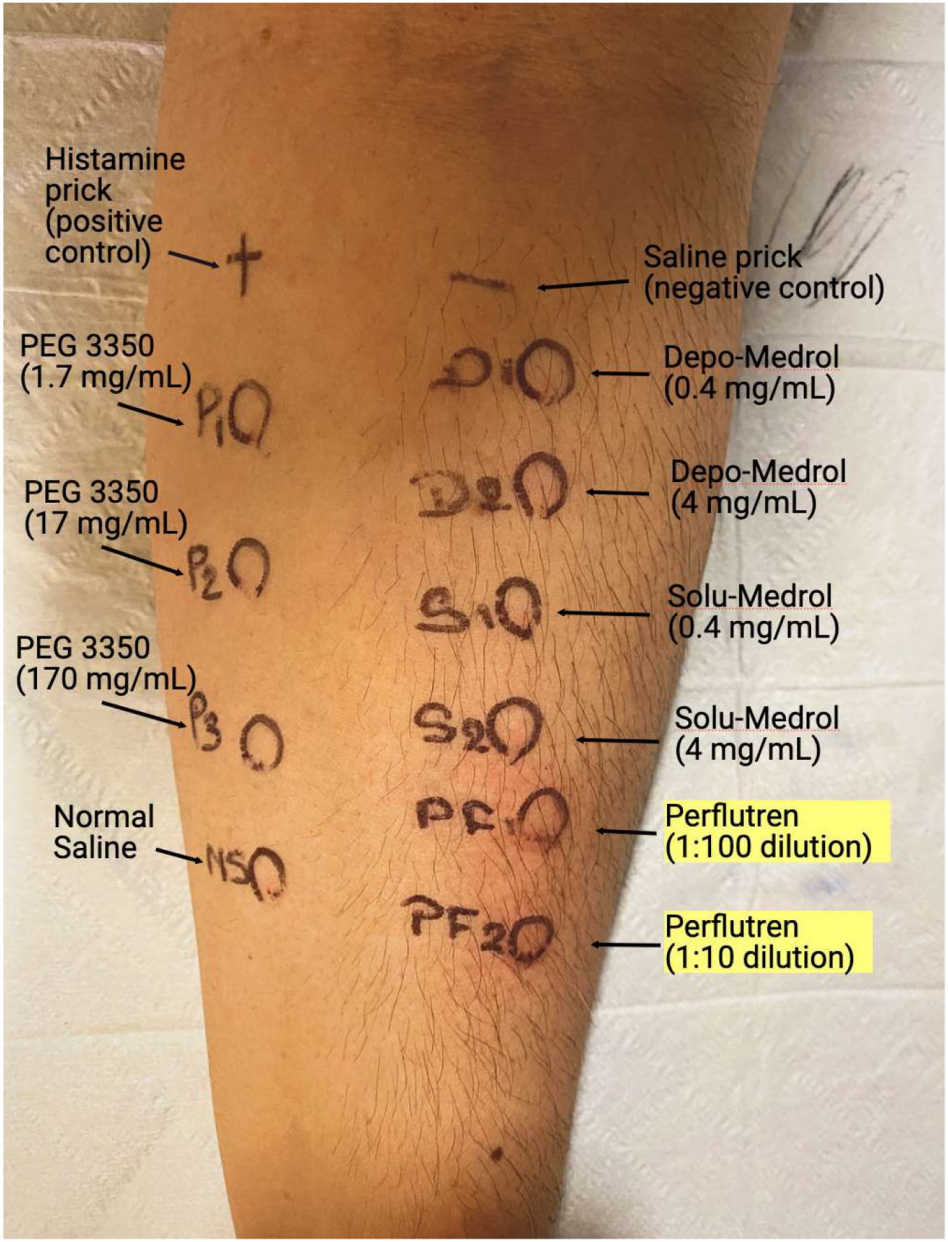
Skin testing results. The patient had appropriate responses to SPT controls. She tested negative for serial dilutions of PEG 3350 SPT. On IDT, she was only positive to perflutren (Definity®; both concentrations) at 15-minute read. On a separate day, SPT to PEG 8000 was negative (results not shown)

The patient came to our drug allergy clinic one month later and underwent skin prick testing (SPT) to PEG 3350 (1.7 mg/mL and 17 mg/mL) and intradermal testing (IDT) to activated Definity® (agitated with Vialmix® apparatus to form a milky-white liquid suspension of octafluoropropane encapsulated by lipid-shell microbubbles; 1:100 and 1:10 dilutions), methylprednisolone acetate (which contains PEG 3350; 0.4 mg/mL and 4 mg/mL) and methylprednisolone sodium succinate (which does not contain PEG 3350, as a control; 0.4 mg/mL and 4 mg/mL). Following negative SPT and IDT to PEG, she proceeded to a two-step oral challenge with PEG 3350 to a cumulative dose of 17 g. She remained under observation for more than 1 h without evidence of an immediate allergic reaction. However, the patient had a positive IDT to Definity® at both dilutions, with the injection sites becoming indurated with surrounding erythema and expanding outside the IDT sites 15 min after administration (Fig. 1). Definity® IDT at both concentrations were also administered in two controls with negative outcomes, decreasing the possibility of an irritant effect. On a separate day, she underwent SPT to PEG 8000 (5 g dissolved in 10 mL saline) with negative results. Based on the testing outcomes, we concluded that she had an IgE-mediated reaction to a non-PEGylated component of Definity®.

## Discussion and conclusions

Polyethylene glycols, also known as macrogols, are hydrophilic molecules that are often used in medical, pharmaceutical, and cosmetic products. They are available in a range of molecular weights from 200 to 35,000 g/mol with PEG 3350 as a common ingredient in laxatives and bowel preparations. PEG derivatives are structurally similar polymers and are often used as drug excipients [5], as is the case for Definity® microspheres. Many severe immediate-type hypersensitivity reactions have been reported following the use of PEG 4000, 6000, 8000 and 20,000, [6] and in reports between 1989 and 2017, the US FDA identified 53 cases of anaphylactic reactions to bowel preparations or laxatives containing PEG [5]. Anaphylaxis to PEGylated UCAs have been reported in at least 11 cases of patients with prior PEG allergy [7]. However, reports specifically on Definity® have been rare overall. In a retrospective analysis, more than 66,000 administered doses of Definity® over a period of 4.5 ± 2.4 years were analyzed, of which only 8 serious adverse events were reported as probably related to Definity®. Thus, the incidence of anaphylaxis to Definity® was recorded to be 0.006% [3], although confirmatory allergy testing was not performed; thus, the mechanism(s) of the reactions were not determined.

To our knowledge, there is only one case report of an anaphylactic reaction to Definity® clearly attributed to the PEGylated part of the microsphere. The patient was a 58-year-old male who, within minutes of being given Definity® for a stress myocardial perfusion scan, developed urticaria, shortness of breath, and hypotension. Previously, he had developed swollen hands when handling paints containing high molecular weight PEG and anaphylaxis to oral PEG 3350 during preparation for a colonoscopy. He later tested positive on SPT to PEG 3350 and methylprednisolone acetate (containing PEG), and positive on IDT to triamcinolone acetonide (containing polysorbate 80). It was concluded that his immediate reactions to the PEG products and Definity® were due to an IgE-mediated reaction to PEG [4].

On the other hand, our patient had positive IDTs to Definity® but not to PEG-containing medications, and tolerated an oral challenge to PEG 3350. She also tested negative for PEG 8000 SPT, deeming the higher molecular weight PEG 5750 in Definity® to be an unlikely culprit. Definity® contains excipients in the buffer such as glycerin and propylene glycol (Table 1) which are often considered immunologically inert. Other excipients in Definity®, including sodium phosphate dibasic heptahydrate and sodium phosphate monobasic monohydrate (Table 1) can also be found in methylprednisolone sodium succinate [8], to which the patient tested negative. Chlorhexidine was not used as an antiseptic and the patient has no history of latex or chlorhexidine allergy, thus she was not tested for these allergens. In addition, our patient had exercise as a cofactor, which could have contributed to the severe presentation, but she generally tolerates physical activity.

**Table 1:** Composition of Definity® [2]

Core gas	Shell composition	Excipients
Octafluoropropane	• 1,2-dipalmitoyl-sn-glycero-3-phosphatidic acid, monosodium salt (DPPA) • 1,2-dipalmitoyl-sn-glycero-3-phosphatidylcholine (DPPC) • N-(methoxypolyethylene glycol 5000 carbamoyl)-1,2-dipalmitoyl-sn-glycero-3-phosphatidylethanolamine, monosodium salt (MPEG5000 DPPE)	• Propylene glycol • Glycerin • Sodium phosphate monobasic monohydrate • Sodium phosphate dibasic heptahydrate • Sodium chloride • Water

An alternative explanation for the patient’s reaction could be a complement activation-related pseudoallergy (CARPA). The mechanism of UCA clearance in the bloodstream is through opsonization via activation of the complement system [7]. In addition, since many symptoms of CARPA are similar to IgE-mediated reactions, it is difficult to conclude the causative mechanism behind our patient’s anaphylaxis. In the retrospective analysis by Wei et al., 4 of the reactions to Definity were described as anaphylactiod and compatible with CARPA, with symptoms including hypotension, dyspnea, urticaria, and swelling of the face and throat. They all occurred within 15 min of administration and patients recovered after 4 to 8 h in the hospital after being treated with fluids, antihistamines, steroids, and/or epinephrine [3].

In summary, immediate hypersensitivity reactions to Definity® are very rare, with most reported cases attributing PEG as the likely allergen. We present the first documented case of anaphylaxis potentially triggered by a non-PEG component of Definity®. A plausible explanation could be a CARPA reaction. However, based on negative PEG testing results and positive IDTs to Definity®, we suspect the reaction was an IgE-mediated response to the non-PEGylated component of Definity®. This case highlights a very rare case of anaphylaxis to Definity® unrelated to the PEG excipient and the importance of physicians to be aware of this rare but critical situation.

## Data Availability

The datasets used and/or analysed during the current study are available from the corresponding author on reasonable request.

## Abbreviations

UCA: Ultrasound contrast agent
MW: Molecular weight
PEG: Polyethylene glycol
SPT: Skin prick test
IDT: Intradermal test
CARPA: Complement activation-related pseudoallergy
